# Association of NFE2L2 Gene Polymorphisms with Risk and Clinical Characteristics of Acute Type A Aortic Dissection in Han Chinese Population

**DOI:** 10.1155/2021/5173190

**Published:** 2021-07-17

**Authors:** Yiran Zhang, Qi Zheng, Ruoshi Chen, Xiaoyi Dai, Yimin Zhu, Liang Ma

**Affiliations:** ^1^Department of Cardiovascular Surgery, First Affiliated Hospital, School of Medicine, Zhejiang University, Hangzhou 310003, China; ^2^Department of Epidemiology & Biostatistics, School of Public Health, Zhejiang University, Hangzhou 310058, China

## Abstract

The present study is aimed at investigating the association of NFE2L2 gene polymorphisms with risk and clinical characteristics of acute type A aortic dissection (AAAD) in a Han Chinese population. Six SNPs (rs1806649, rs13001694, rs2364723, rs35652124, rs6721961, and rs2706110) in NFE2L2 were genotyped using SNaPshot Multiplex Kit in 94 adult patients diagnosed with AAAD at our hospital, and 208 healthy Han Chinese subjects from the 1000 Genomes Project were served as the control group. The CC genotype of rs2364723 (CC versus (GC+GG), OR = 2.069, 95% CI: 1.222-3.502, *p* = 0.006) and CC genotype of rs35652124 (CC versus (CT+TT), OR = 1.889, 95% CI: 1.112-3.210, *p* = 0.018) were identified as risk factors for AAAD. Multivariable linear regression analysis revealed that the CC genotype of rs2364723 (*β* = 5.031, 95% CI: 1.878-8.183, *p* = 0.002) and CC genotype of rs35652124 (*β* = 4.751, 95% CI: 1.544-7.958, *p* = 0.004) were associated with increased maximum ascending aorta diameter of AAAD. Patients carrying rs2364723 CC genotype had a higher incidence of coronary artery involvement (31% vs. 12%, *p* = 0.027), while patients carrying rs35652124 CC genotype had a higher incidence of brain ischemia (9% vs. 0%, *p* = 0.045). In conclusion, NFE2L2 gene polymorphisms were correlated with risk and severity of AAAD in Han Chinese population.

## 1. Introduction

Acute type A aortic dissection (AAAD) is a medical emergency with 48.6% of the patients died before hospital admission [1] and in-hospital mortality rate as high as 32.5% [2]. Therefore, it is important to identify the pathogenesis and risk factors of AAAD, which could help prevention and early intervention of the disease.

Genetic susceptibility is an important risk factor for AAAD. It has been shown that nearly 20% AAAD patients also had hereditary disorders such as Marfan syndrome, Loeys-Dietz syndrome, and Turner syndrome [3]. Several recent studies reported that polymorphisms in genes such as TLR4 [4], ALDH2 [5], and Mfn2 [6] were associated with risk of sporadic aortic dissection. However, the genetic determinants of AAAD remained largely undiscovered.

It has been indicated that oxidative stress participated in degeneration and necrosis of aortic media, which played an essential role in the development of aortic dissection. A proteomic study showed that the expression and activity of superoxide dismutase were decreased in the aortic media of patients with thoracic aortic dissection, while the level of lipid peroxidation was increased [7]. It has been shown that increased fluid shear stress on aorta wall led to accumulation of reactive oxygen species (ROS), inducing phenotype switch of vascular smooth muscle cells, which played a vital role in formation of aortic aneurysm and dissection [8, 9].

Nuclear factor erythroid 2 like 2 (NFE2L2) is a gene localized on chromosome 2q31.2, which encodes a transcription factor Nrf2. Nrf2 regulates a number of antioxidative genes by binding to antioxidant response elements (ARE) in their promoters, and the Nrf2-ARE signaling played a protective role in various kinds of oxidative stress injury [10]. A number of studies suggested that activating Nrf2-ARE signaling could alleviate apoptosis [11], calcification [12], and phenotype switch [13] of vascular smooth muscle cells. Moreover, several single-nucleotide polymorphisms (SNPs) in NFE2L2 gene have been reported to be associated with coronary artery disease [14], vascular stiffness [15], and cardiovascular mortality [16] in different populations. Thus, we inferred that NFE2L2 gene polymorphisms may be associated with AAAD, while no study has examined this relationship. The present study investigated the association of NFE2L2 gene polymorphisms with risk and clinical characteristics of AAAD in a Han Chinese population.

## 2. Materials and Methods

### 2.1. Study Population

**Figure 1:**
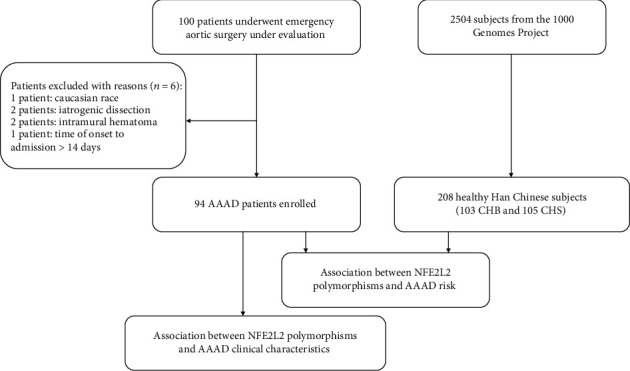
Flow chart of the study design. AAAD: acute type A aortic dissection; CHB: Han Chinese in Beijing, China; CHS: southern Han Chinese, China.

This study was approved by the ethics committee of the First Affiliated Hospital, School of Medicine, Zhejiang University in China (reference number: IIT2020-277). All participants gave informed consents before inclusion in the study. The inclusion criteria were as follows: (1) Han Chinese patients older than 18 years, (2) aortic dissection diagnosed according to computed tomography angiography (CTA) and echocardiography, and (3) the time from the onset of the symptom to admission within 14 days. The exclusion criteria were as follows: (1) patients with familial aortic dissection or other types of acute aortic syndrome (i.e., intramural hematoma, penetrating ulcer, and iatrogenic/traumatic dissection) and (2) patients who did not sign the informed consents. A total of 94 adult patients diagnosed with AAAD were enrolled in this study between September 2018 and November 2020 at our hospital (Figure 1).

5 mL of the peripheral blood was collected from each participant and stored at -80°C for further examination. All blood samples were handled anonymously. Clinical variables of each patient were obtained through review of medical records, which included demographics (age and gender), medical histories (hypertension, diabetes, chronic kidney disease, prior aortic stent implant, prior cardiac surgery, smoking, and bicuspid aortic valve), blood pressure on admission (systolic and diastolic), maximum diameter of ascending aorta (measured by echocardiography preoperatively), severity of aortic valve regurgitation, the site and number of primary/secondary entry tears (according to the operation note), DeBakey classification of the dissection, organ ischemia (brain, coronary artery involvement, and lower limber), and laboratory tests on admission (white blood cell count, platelet count, hemoglobin, high sensitivity C-reactive protein, fibrinogen, D-dimer, serum uric acid, creatine, and urea nitrogen).

The control group consisted of 208 healthy Han Chinese subjects from the publicly available population-based database of the 1000 Genomes Project, which included 103 Han Chinese in Beijing, China (CHB) and 105 Southern Han Chinese, China (CHS) (Figure 1).

### 2.2. SNP Selection and Genotyping

Six SNPs (rs1806649 [17], rs13001694 [16], rs2364723 [16], rs35652124 [18], rs6721961 [14, 15], and rs2706110 [19]) located in NFE2L2 previously reported to be associated with cardiovascular risk were selected for genotyping.

Genomic DNA was extracted from venous blood samples using Trelief Animal Genomic DNA Kit (Beijing TsingKe Biotech Co., Ltd., Beijing, China) according to the manufacturer's instruction. Genotyping of the six SNPs was performed using the SNaPshot Multiplex Kit (Thermo Fisher Scientific, Waltham, MA, USA). Amplification primers and extension primers are listed in Table 1. Briefly, multiplex polymerase chain reaction was performed followed by a single-nucleotide primer extension assay test. The SNPs were detected by capillary electrophoresis using ABI 3730xl DNA Analyzer (Thermo Fisher Scientific, Waltham, MA, USA). The data was analyzed using GeneMapper 4.1 software (Applied Biosystems, Foster City, CA, USA).

### 2.3. Statistical Analysis

The Hardy-Weinberg equilibrium (HWE) was evaluated using Genetics package in R software to determine the representativeness of the study population. Categorical variables were presented as numbers and proportions and were compared between different groups using Pearson's chi-square or Fisher's exact test, as appropriate. Normally distributed continuous data were presented as the mean ± SD and were compared using unpaired Student *t*-test. Nonnormally distributed continuous data were presented as median with interquartile range (IQR) and were compared by the Mann-Whitney *U* test. Dominant, recessive, and homozygote genetic models of inheritance were chosen to evaluate the association between each SNP and AAAD risk with odds ratio (OR) and 95% confidence interval (95% CI). Multivariable linear regression model was used to adjust the effect of potential confounders on the association between NFE2L2 gene polymorphisms and maximum ascending aorta diameter. All statistical analyses were processed using SPSS 25.0 software (SPSS Inc., Chicago, IL, USA) and R programming language (version 4.0.0). All *p* values of <0.05 were considered statistically significant.

## 3. Results

### 3.1. Genotype and Allele Frequencies of NFE2L2 Gene Polymorphisms

The genotype frequencies for all 6 SNPs were in line with HWE in the control group (rs1806649, *p* = 0.379; rs13001694, *p* = 0.741; rs2364723, *p* = 1.000; rs35652124, *p* = 1.000; rs6721961, *p* = 0.600; and rs2706110, *p* = 0.450) and the AAAD group (rs1806649, *p* = 1.000; rs13001694, *p* = 1.000; rs2364723, *p* = 0.292; rs35652124, *p* = 0.285; rs6721961, *p* = 0.205; and rs2706110, *p* = 0.754), indicating that the results of the present study have a representative genetic group. The genotype distribution and allele frequency of each SNP are shown in Tables 2 and 3. The genotype distribution of rs2364723 differed significantly between the AAAD group and control group (*p* = 0.021). The allele frequencies of rs2364723 (*p* = 0.009) and rs35652124 (*p* = 0.021) were significantly linked to AAAD risk.

### 3.2. Association between NFE2L2 Gene Polymorphisms and AAAD Risk

The association between NFE2L2 gene polymorphisms and AAAD risk in different genetic models is shown in Table 4. rs2364723 was strongly correlated with the risk of AAAD in the recessive genetic model (CC versus (GC+GG), OR = 2.069, 95% CI: 1.222-3.502, *p* = 0.006) and the homozygous model (CC versus GG, OR = 2.333, 95% CI: 1.177-4.627, *p* = 0.014), which indicated that the CC genotype of rs2364723 was a risk factor for AAAD. rs35652124 was strongly correlated with the risk of AAAD in the recessive genetic model (CC versus (CT+TT), OR = 1.889, 95% CI: 1.112-3.210, *p* = 0.018) and the homozygous model (CC versus TT, OR = 2.125, 95% CI: 1.076-4.195, *p* = 0.029), which indicated that the CC genotype of rs35652124 was a risk factor for AAAD.

### 3.3. Association between rs2364723 and rs35652124 Polymorphisms with AAAD Clinical Characteristics

**Figure 2:**
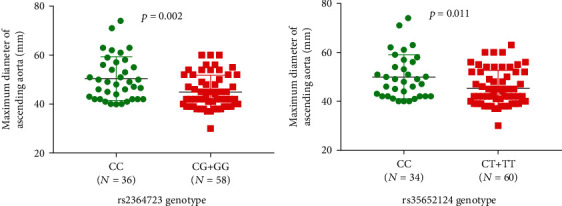
Maximum diameter of ascending aorta in AAAD patients grouped by rs2364723 and rs35652124 genotypes.

Table 5 summarizes the clinical characteristics of the 94 AAAD patients in different genotype groups. Compared with patients carrying rs2364723 GC+GG genotypes, patients with rs2364723 CC genotype had increased maximum diameter of ascending aorta (median diameter, 49 vs. 42 mm, *p* = 0.002, Table 5 and Figure 2(a)) and higher incidence of coronary artery involvement (31% vs. 12%, *p* = 0.027, Table 5). Compared with patients carrying rs35652124 CT+TT genotypes, patients with rs35652124 CC genotype had increased maximum diameter of ascending aorta (median diameter, 49 vs. 43 mm, *p* = 0.011, Table 5 and Figure 2(b)) and higher incidence of brain ischemia (9% vs. 0%, *p* = 0.045, Table 5).

### 3.4. Multivariable Linear Regression Analysis for Maximum Ascending Aorta Diameter

After adjusting for potential confounders including demographics (age and gender) and medical histories (hypertension, diabetes, chronic kidney disease, prior aortic stent implant, prior cardiac surgery, smoking, and bicuspid aortic valve) using multivariable linear regression model, we found that rs2364723 (Model 1, *β* = 5.031, 95% CI: 1.878-8.183, *p* = 0.002, Table 6) and rs35652124 (Model 2, *β* = 4.751, 95% CI: 1.544-7.958, *p* = 0.004, Table 6) were still significantly associated with maximum ascending aorta diameter. Besides, prior cardiac surgery was also found to be significantly associated with maximum ascending aorta diameter (Model 1, *β* = 9.652, 95% CI: 2.142-17.162, *p* = 0.012; Model 2, *β* = 10.228, 95% CI: 2.646-17.810, *p* = 0.009, Table 6).

## 4. Discussion

The present study was the first to evaluate the association of NFE2L2 gene polymorphisms with risk and clinical characteristics of AAAD. Our data indicated that the CC genotype of rs2364723 and CC genotype of rs35652124 were risk factors for AAAD. Multivariable linear regression analysis revealed that the CC genotype of rs2364723 and CC genotype of rs35652124 were associated with increased maximum ascending aorta diameter. Besides, patients carrying rs2364723 CC genotype had a higher incidence of coronary artery involvement, while patients carrying rs35652124 CC genotype had a higher incidence of brain ischemia.

The protective role of Nrf2 on aortic dissection and aneurysm has been indicated by several studies. It has been shown that the Nrf2 expression in the thoracic aortic aneurysm tissue from patients with Loeys-Dietz Syndrome was decreased, compared with nondamaged aortic tissue from control subjects [20]. Animal experiments using angiotensin II-induced aortic aneurysm model in mice showed that NFE2L2 gene deficiency increased the risk of development and rupture of aortic aneurysm [21, 22]. The potential mechanisms underlying the protective role of Nrf2 on aortic dissection and aneurysm may be multifactorial. It has been suggested that activating the Nrf2-ARE pathway suppressed the angiotensin II-induced phenotype switch and proliferation [13], apoptosis [11], calcification [12], and fibrotic [23] of vascular smooth muscle cells. It has also been found that activating the Nrf2-ARE pathway promoted endothelial repair during vascular injury [24].

In the present study, we found that CC genotype of rs2364723 and CC genotype of rs35652124 were linked to an increased risk of AAAD. The rs35652124 polymorphism locates in the promoter region of the NFE2L2 gene, which might regulate Nrf2 expression by influencing the NFE2L2 promoter activity [25]. In vitro transient transfection reporter gene assays showed that rs35652124 C allele reduced the NFE2L2 promoter activity in human microvascular endothelial cells [18]. It has been found that, in healthy African American population, rs35652124 C allele carriers had lower forearm blood flow (FBF) and higher forearm vascular resistance (FVR) under basal condition as well as in response to vasodilators [18]. The maximum FBF response to endothelium-dependent vasodilator in patients with Marfan syndrome has been found to be significantly reduced [26]. These evidences indicate that NFE2L2 gene polymorphisms were associated with vasomotion abnormality, which might play a role in aortic enlargement and dissection. The rs2364723 polymorphism locates in the intron 1 region of the NFE2L2 gene. In a general population-based cohort of Caucasian, it was found that rs2364723 GG carriers had lower cardiovascular mortality and triglyceride levels compared with GC+CC carriers [16]. While abnormal serum lipid composition has been indicated to play a key role in development of aortic dissection [27], it should be noted that the rs2364723 polymorphism was in high linkage disequilibrium (LD) with rs35652124 (*r*^2^ = 0.99) [28]; thus, the association of rs2364723 with AAAD risk might also represent the effect of rs35652124 due to almost complete LD.

We further investigated the correlation of rs2364723 and rs35652124 polymorphisms with clinical characteristics of AAAD patients and found that rs2364723 CC carriers and rs35652124 CC carriers had increased maximum ascending aorta diameter. Increased ascending aorta diameter is associated with reduced elastin density and increased collagen density, which has a profound impact on aortic dissection [29]. A study on 230 patients with thoracic aortic aneurysm from 1985 to 1996 showed that the median diameter of ascending aorta was 6.0 cm at time of aorta rupture or dissection [30]. More recent data on nature history of ascending aortic aneurysm between 5 and 6 cm found two hinge points for aorta rupture or dissection—one at 5.25 cm and the other at 5.75 cm [31]. Recent studies also indicated that aortic diameter ≥ 40 mm was an independent risk factor for in-hospital mortality of acute aortic dissection [32], and increased ascending aortic diameter was found to be negatively correlated with total survival time in patients with ascending aortic dissection [33]. Our study also found the correlation of rs2364723 and rs35652124 polymorphisms with incidence of organ ischemia such as brain ischemia and coronary artery involvement. Recent data showed that vital organ ischemia was a risk factor for early mortality after AAAD repair [34]. These evidences suggested that NFE2L2 gene polymorphisms not only correlated with onset risk but also severity of AAAD.

There are several limitations in the present study. Firstly, it is a pilot, single-center research with relatively small sample size, and our findings cannot be generalized to the general population since we included only Han Chinese subjects. Secondly, this study only focused on 6 SNPs previously reported to be associated with cardiovascular risk, while other SNPs with potential influence were not examined. Moreover, we had not collected the aortic tissue of the AAAD patients, which made us unable to measure the oxidative stress condition in dissected aorta of the patients. Thus, further multicenter studies with different ethnicities and larger sample size were needed, and additional studies should determine the mechanisms underlying the effects of gene polymorphisms on functions of NFE2L2, and its role in the pathogenesis of AAAD.

## 5. Conclusions

The present study showed that rs2364723 and rs35652124 polymorphisms in NFE2L2 gene were correlated with risk of AAAD in a Han Chinese population. These two SNPs were also related to maximum ascending aorta diameter and vital organ ischemia in AAAD patients.

## Figures and Tables

**Table 1:** Amplification primers and extension primers of selected SNPs.

SNPs	Amplification primers	Extension primers
rs1806649	Forward: ACTGTCACAGCATTGTCATGTATCA	TTTTTTTTTTTTTTAGTCTTAGAGGAACTCATATCCTAAG
	Reverse: TAGGAATCTTTCAGGGCATGAAG	
rs13001694	Forward: TTGCAGCTGTAGGTCCGAGA	TTTTTTTTTTTTTCTGGACAAGTCACTCTACCTGC
	Reverse: TTTTGTAGGGAAGCCTGCCA	
rs2364723	Forward: AGCAATTATGTCTGGCACCC	TTTTTTTTTTTTTTTTTTTTTTTGGTTTTGTGTCAATATTTCCTC
	Reverse: CCAGGAAAAGTTGAAAGCCAAATG	
rs35652124	Forward: CTCACTTTACCGCCCGAGAA	TTTTTTTTTTTTTTTTTTTTTTTTTTTTTTACACGTGGGAGTTCAGAGG
	Reverse: GTTCTCTTGGGGTTCCCGTT	
rs6721961	Forward: CTCACTTTACCGCCCGAGAA	TTTTTTTTTTTTTTTTTTTTTTTTTTTTTTTTTCTAGGGGAGATGTGGACAGC
	Reverse: GTTCTCTTGGGGTTCCCGTT	
rs2706110	Forward: GCTCCCCTCAAAAACAGGAAC	TTTTTTTTTTTTTTTTTTTTTTTTTTTTTTTTTTTATTAGTCATGGCATAGTTGAGA
	Reverse: ACGAGGGAACTGGCCATAGAA	

**Table 2:** Genotype distribution of NFE2L2.

SNP		Genotype (frequency, %)			*χ* ^2^	*p* value
		TT	CT	CC		
rs1806649	Case	1 (1.1)	17 (18.1)	76 (80.9)	0.289	0.865
	Control	3 (1.4)	33 (15.9)	172 (82.7)		
		GG	GA	AA		
rs13001694	Case	2 (2.1)	22 (23.4)	70 (74.5)	0.311	0.856
	Control	4 (1.9)	43 (20.7)	161 (77.4)		
		CC	GC	GG		
rs2364723	Case	36 (38.3)	40 (42.6)	18 (19.1)	7.742	0.021
	Control	48 (23.1)	104 (50.0)	56 (26.9)		
		CC	CT	TT		
rs35652124	Case	34 (36.2)	41 (43.6)	19 (20.2)	5.891	0.053
	Control	48 (23.1)	103 (49.5)	57 (27.4)		
		TT	GT	GG		
rs6721961	Case	2 (2.1)	35 (37.2)	57 (60.6)	4.434	0.109
	Control	15 (7.2)	87 (41.8)	106 (51.0)		
		TT	CT	CC		
rs2706110	Case	3 (3.2)	32 (34.0)	59 (62.8)	1.005	0.605
	Control	10 (4.8)	79 (38.0)	119 (57.2)		

**Table 3:** Allele frequency of NFE2L2.

SNP		Allele (frequency, %)		*χ* ^2^	*p* value
		T	C		
rs1806649	Case	19 (10.1)	169 (89.9)	0.080	0.778
	Control	39 (9.4)	377 (90.6)		
		G	A		
rs13001694	Case	26 (13.8)	162 (86.2)	0.287	0.592
	Control	51 (12.3)	365 (87.7)		
		C	G		
rs2364723	Case	112 (59.6)	76 (40.4)	6.854	0.009
	Control	200 (48.1)	216 (51.9)		
		C	T		
rs35652124	Case	109 (58.0)	79 (42.0)	5.330	0.021
	Control	199 (47.8)	217 (52.2)		
		T	G		
rs6721961	Case	39 (20.7)	149 (79.3)	3.682	0.055
	Control	117 (28.1)	299 (71.9)		
		T	C		
rs2706110	Case	38 (20.2)	150 (79.8)	0.949	0.330
	Control	99 (23.8)	317 (76.2)		

**Table 4:** Association of NFE2L2 gene polymorphisms with risk of AAAD.

SNP	Genetic model	Genotype	OR (95% CI)	*p* value
rs1806649	Dominant	(TT+CT) vs. CC	1.132 (0.605-2.118)	0.699
	Recessive	TT vs. (CT+CC)	0.735 (0.075-7.158)	1.000
	Homozygote	TT vs. CC	0.754 (0.077-7.370)	1.000
rs13001694	Dominant	(GG+GA) vs. AA	1.174 (0.667-2.069)	0.578
	Recessive	GG vs. (GA+AA)	1.109 (0.199-6.161)	1.000
	Homozygote	GG vs. AA	1.150 (0.206-6.425)	1.000
rs2364723	Dominant	(CC+GC) vs. GG	1.556 (0.855-2.829)	0.146
	Recessive	CC vs. (GC+GG)	2.069 (1.222-3.502)	0.006
	Homozygote	CC vs. GG	2.333 (1.177-4.627)	0.014
rs35652124	Dominant	(CC+CT) vs. TT	1.490 (0.827-2.684)	0.182
	Recessive	CC vs. (CT+TT)	1.889 (1.112-3.210)	0.018
	Homozygote	CC vs. TT	2.125 (1.076-4.195)	0.029
rs6721961	Dominant	(TT+GT) vs. GG	0.675 (0.411-1.107)	0.118
	Recessive	TT vs. (GT+GG)	0.280 (0.063-1.249)	0.076
	Homozygote	TT vs. GG	0.248 (0.055-1.123)	0.052
rs2706110	Dominant	(TT+CT) vs. CC	0.793 (0.481-1.308)	0.364
	Recessive	TT vs. (CT+CC)	0.653 (0.175-2.429)	0.738
	Homozygote	TT vs. CC	0.605 (0.160-2.282)	0.659

**Table 5:** Association of rs2364723 and rs35652124 polymorphisms with AAAD clinical characteristics.

Variables	Overall	rs2364723			rs35652124		
		CC	GC or GG	*p*	CC	CT or TT	*p*
Number of patients	94	36	58		34	60	
Demographics							
Age (years)	52 ± 13	52 ± 13	52 ± 13	0.865	51 ± 13	52 ± 13	0.762
Male, *n* (%)	75 (80)	30 (83)	45 (78)	0.500	28 (82)	47 (78)	0.641
Medical histories, *n* (%)							
Hypertension	63 (67)	25 (69)	38 (66)	0.694	24 (71)	39 (65)	0.580
Diabetes	3 (3)	2 (6)	1 (2)	0.556	1 (3)	2 (3)	1.000
CKD	3 (3)	1 (3)	2 (3)	1.000	1 (3)	2 (3)	1.000
Prior aortic stent implant	3 (3)	1 (3)	2 (3)	1.000	1 (3)	2 (3)	1.000
Prior cardiac surgery	5 (5)	2 (6)	3 (5)	1.000	1 (3)	4 (7)	0.650
Smoking	38 (40)	16 (44)	22 (38)	0.532	15 (44)	23 (38)	0.583
Bicuspid aortic valve	7 (7)	5 (14)	2 (3)	0.102	5 (15)	2 (3)	0.094
Clinical presentation							
Systolic blood pressure (mmHg)	151 ± 27	152 ± 23	151 ± 30	0.817	151 ± 22	152 ± 30	0.870
Diastolic blood pressure (mmHg)	82 ± 19	78 ± 17	84 ± 20	0.127	77 ± 17	84 ± 20	0.075
AoD_max_ (mm)	45 (41-52)	49 (42-57)	42 (39-51)	0.002	49 (42-56)	43 (39-52)	0.011
Organ ischemia, *n* (%)							
Brain	3 (3)	3 (8)	0 (0)	0.053	3 (9)	0 (0)	0.045
Lower limb	15 (16)	8 (22)	7 (12)	0.191	6 (18)	9 (15)	0.736
Coronary artery involvement	18 (19)	11 (31)	7 (12)	0.027	10 (29)	8 (13)	0.057
Dissection characteristics							
DeBakey classification, *n* (%)							
Type I	84 (89)	32 (89)	52 (90)	1.000	30 (88)	54 (90)	1.000
Type II	10 (11)	4 (11)	6 (10)		4 (12)	6 (10)	
Primary entry site, *n* (%)							
Ascending aorta	62 (66)	26 (72)	36 (62)	0.600	25 (74)	37 (62)	0.490
Aortic arch	19 (20)	6 (17)	13 (22)		5 (15)	14 (23)	
Descending aorta or unknown	13 (14)	4 (11)	9 (16)		4 (12)	9 (15)	
Secondary entry site, *n* (%)							
None	76 (81)	29 (81)	47 (81)	1.000	28 (82)	48 (80)	0.948
Ascending aorta	2 (2)	1 (3)	1 (2)		1 (3)	1 (2)	
Aortic arch	14 (15)	5 (14)	9 (16)		4 (12)	10 (17)	
Descending aorta	2 (2)	1 (3)	1 (2)		1 (3)	1 (2)	
Entry tears≥2, n (%)	18 (19)	7 (19)	11 (19)	0.954	6 (18)	12 (20)	0.781
Aortic valve regurgitation, *n* (%)							
None	14 (15)	4 (11)	10 (17)	0.298	3 (9)	11 (18)	0.205
Mild	36 (38)	14 (39)	22 (38)		13 (38)	23 (38)	
Mild-moderate	11 (12)	3 (8)	8 (14)		3 (9)	8 (13)	
Moderate	14 (15)	7 (19)	7 (12)		7 (21)	7 (12)	
Moderate-severe	10 (11)	2 (6)	8 (14)		2 (6)	8 (13)	
Severe	9 (10)	6 (17)	3 (5)		6 (18)	3 (5)	
Laboratory tests on admission							
WBC (10^9^/L)	13.2 ± 4.0	13.1 ± 4.1	13.3 ± 4.9	0.904	13.1 ± 4.3	13.3 ± 3.8	0.878
PLT (10^9^/L)	156 (124-192)	155 (137-184)	157 (122-208)	0.800	154 (137-183)	157 (122-211)	0.620
Hb (g/L)	134 ± 22	133 ± 24	134 ± 20	0.957	134 ± 24	133 ± 20	0.831
CRP (mg/L)	6.8 (2.4-20.3)	8.7 (2.7-19.2)	5.3 (2.3-22.1)	0.602	8.7 (2.7-21.3)	5.6 (2.3-20.9)	0.698
Fibrinogen (g/L)	2.13 (1.52-2.79)	2.36 (1.47-3.03)	1.95 (1.52-2.64)	0.621	2.31 (1.45-3.00)	1.96 (1.53-2.74)	0.953
D-dimer (*μ*g/mL FEU)	10.9 (4.8-28.6)	8.9 (4.2-27.2)	12.0 (5.8-29.8)	0.283	9.6 (4.2-29.9)	11.4 (5.7-27.5)	0.544
UA (*μ*mol/L)	405 ± 110	397 ± 115	411 ± 107	0.574	402 ± 116	407 ± 107	0.831
Creatinine (*μ*mol/L)	98 (76-127)	98 (78-129)	97 (74-127)	0.876	96 (77-124)	98 (75-128)	0.642
BUN (mmol/L)	6.78 (5.39-7.98)	6.56 (5.36-7.87)	6.91 (5.49-8.07)	0.735	6.56 (5.38-7.68)	6.91 (5.41-8.12)	0.645

Data are represented as the median (interquartile range), *n*(%), or mean ± SD. CKD: chronic kidney disease; AoD_max_: maximum diameter of ascending aorta; WBC: white blood cell count; PLT: platelet; Hb: hemoglobin; UA: uric acid; BUN: blood urea nitrogen.

**Table 6:** Multiple linear regression of maximum ascending aorta diameter.

Model 1				Model 2			
Variables	*β* (95% CI)	*p* value	Tolerance	Variables	*β* (95% CI)	*p* value	Tolerance
rs2364723 (CC: 1, GC+GG: 0)	5.031 (1.878-8.183)	0.002	0.943	rs35652124 (CC: 1, CT+TT: 0)	4.751 (1.544-7.958)	0.004	0.946
Age	0.126 (-0.21-0.274)	0.093	0.624	Age	0.139 (-0.10-0.287)	0.068	0.625
Male	0.710 (-4.117-5.537)	0.771	0.589	Male	1.054 (-3.806-5.913)	0.667	0.590
Hypertension	-2.945 (-6.213-0.323)	0.077	0.938	Hypertension	-3.149 (-6.447-0.149)	0.061	0.935
Diabetes	2.338 (-6.946-11.622)	0.618	0.831	Diabetes	4.093 (-5.196-13.382)	0.383	0.843
CKD	1.383 (-7.946-10.712)	0.769	0.823	CKD	0.632 (-8.762-10.027)	0.894	0.824
Prior aortic stent implant	6.274 (-2.376-14.925)	0.153	0.957	Prior aortic stent implant	6.379 (-2.337-15.096)	0.149	0.957
Prior cardiac surgery	9.652 (2.142-17.162)	0.012	0.779	Prior cardiac surgery	10.228 (2.646-17.810)	0.009	0.776
Smoking	-0.497 (-3.929-2.934)	0.774	0.780	Smoking	-0.553 (-3.992-2.926)	0.760	0.780
Bicuspid aortic valve	4.633 (-1.274-10.540)	0.123	0.920	Bicuspid aortic valve	4.620 (-1.348-10.588)	0.127	0.915

CKD: chronic kidney disease.

## Data Availability

The data used to support the findings of this study are available from the corresponding authors upon request.
